# Molecular imaging of myogenic stem/progenitor cells with [^18^F]-FHBG PET/CT system in SCID mice model of post-infarction heart

**DOI:** 10.1038/s41598-021-98861-5

**Published:** 2021-10-06

**Authors:** Weronika Wargocka-Matuszewska, Katarzyna Fiedorowicz, Anna Rugowska, Karolina Bednarowicz, Agnieszka Zimna, Łukasz Cheda, Paulina Hamankiewicz, Krzysztof Kilian, Michał Fiedorowicz, Monika Drabik, Natalia Rozwadowska, Zbigniew Rogulski, Maciej Kurpisz

**Affiliations:** 1 Faculty of Chemistry, Biological and Chemical Research Centre, University of Warsaw, Żwirki i Wigury 101, 02-089 Warsaw, Poland; 2 Institute of Human Genetics Polish Academy of Science, Strzeszyńska 32, 60-479 Poznan, Poland; 3 Institute of Human Biology and Evolution, Faculty of Biology Adam, Mickiewicz University, Uniwersytetu Poznańskiego 6, 61-614 Poznan, Poland; 4 Heavy Ion Laboratory, University of Warsaw, Pasteura 5A, 02-093 Warsaw, Poland; 5 Mossakowski Medical Research Centre Polish Academy of Science, Pawińskiego 5, 02-106 Warsaw, Poland

**Keywords:** Cell migration, Cellular imaging, Adult stem cells, Heart stem cells, Regeneration, Biochemistry, Chemical biology, Medicinal chemistry, Nuclear chemistry, Cells, Cardiology

## Abstract

Preclinical and clinical studies have shown that stem cells can promote the regeneration of damaged tissues, but therapeutic protocols need better quality control to confirm the location and number of transplanted cells. This study describes in vivo imaging while assessing reporter gene expression by its binding to a radiolabelled molecule to the respective receptor expressed in target cells. Five mice underwent human skeletal muscle-derived stem/progenitor cell (huSkMDS/PC EF1-HSV-TK) intracardial transplantation after induction of myocardial infarction (MI). The metabolic parameters of control and post-infarction stem progenitor cell-implanted mice were monitored using 2-deoxy-18F-fluorodeoxyglucose ([^18^F]-FDG) before and after double promotor/reporter probe imaging with 9-(4-18F-fluoro-3-[hydroxymethyl]butyl)guanine ([^18^F]-FHBG) using positron emission tomography (PET) combined with computed tomography (CT). Standardized uptake values (SUVs) were then calculated based on set regions of interest (ROIs). Experimental animals were euthanized after magnetic resonance imaging (MRI). Molecular [^18^F]-FHBG imaging of myogenic stem/progenitor cells in control and post-infarction mice confirmed the survival and proliferation of transplanted cells, as shown by an increased or stable signal from the PET apparatus throughout the 5 weeks of monitoring. huSkMDS/PC EF1-HSV-TK transplantation improved cardiac metabolic ([^18^F]-FDG with PET) and haemodynamic (MRI) parameters. In vivo PET/CT and MRI revealed that the precise use of a promotor/reporter probe incorporated into stem/progenitor cells may improve non-invasive monitoring of targeted cellular therapy in the cardiovascular system.

## Introduction

Myocardial infarction is the most common fatal symptom of heart ischaemia and a major cause of death worldwide^1^. Currently, cardiovascular diseases are the leading cause of global morbidity and mortality and are responsible for over 17 million deaths every year^2,3^. The survivors suffer from heart dysfunction and resultant disabilities, which reduces their quality of life. The heart demonstrates little capacity for self-regeneration^4^, and after MI, loses its proper functionality. The post-infarction zone induces a process of cardiac remodelling, which results in major histological and morphological changes in both damaged and neighbouring healthy myocardial tissues^5^. Those who survive MI develop a fibrous scar that can have catastrophic consequences, such as dilated post-ischaemic cardiomyopathy and recurrent MIs^6,7^, potentially even leading to cardiac rupture and sudden death^8^. Therefore, it is necessary to develop a prospective novel therapy to facilitate patient recovery after MI^1^.

There is a growing body of evidence that the use of stem/progenitor cells might positively support the function of damaged pathological tissues^9,10^; however, the majority of current methods for monitoring stem cell transplantation are based on post-mortem histological sampling at single time points. Therefore, the goal of our study was to optimize non-invasive imaging of cellular stem/progenitor cell intervention based on an introduced reporter gene.

To control he biodistribution and retention of transplanted cells, we designed a multimodal reporter gene system. In this system, the elongation factor 1 (EF1) promoter controls herpes simplex thymidine kinase (HSV-TK) and *Renilla luciferase* (Renluc) expression^11^. In turn, the CMV promoter drives the expression of the refluorescent marker mCherry and an antibiotic resistance gene (PuroR). Monitoring by sensitive and high-resolution PET was possible due to stable gene expression, which in particular correlates with the number of viable cells^12,13^.

In this study, [^18^F]-FHBG was used as a reporter probe for imaging HSV-TK. The mechanism comprised trapping the cyclic analogues of guanosine because of the phosphorylation catalysed by the HSV-TK protein product^14^ with consequent visible accumulation in tissues expressing HSV-TK^15^. Using a dedicated small-animal PET probe for the reporter gene, we achieved non-invasive imaging of huSkMDS/PCs EF1-HSV-TK transplanted to the healthy and post-infarction myocardium. To monitor the effects of cellular therapy, we also performed cardiac viability measurements with MRI and quantitative evaluation of murine heart metabolism (viability) through the uptake of [^18^F]-FDG assessed by PET.

## Results

### Cells immunophenotypic characterization

**Figure 1:**
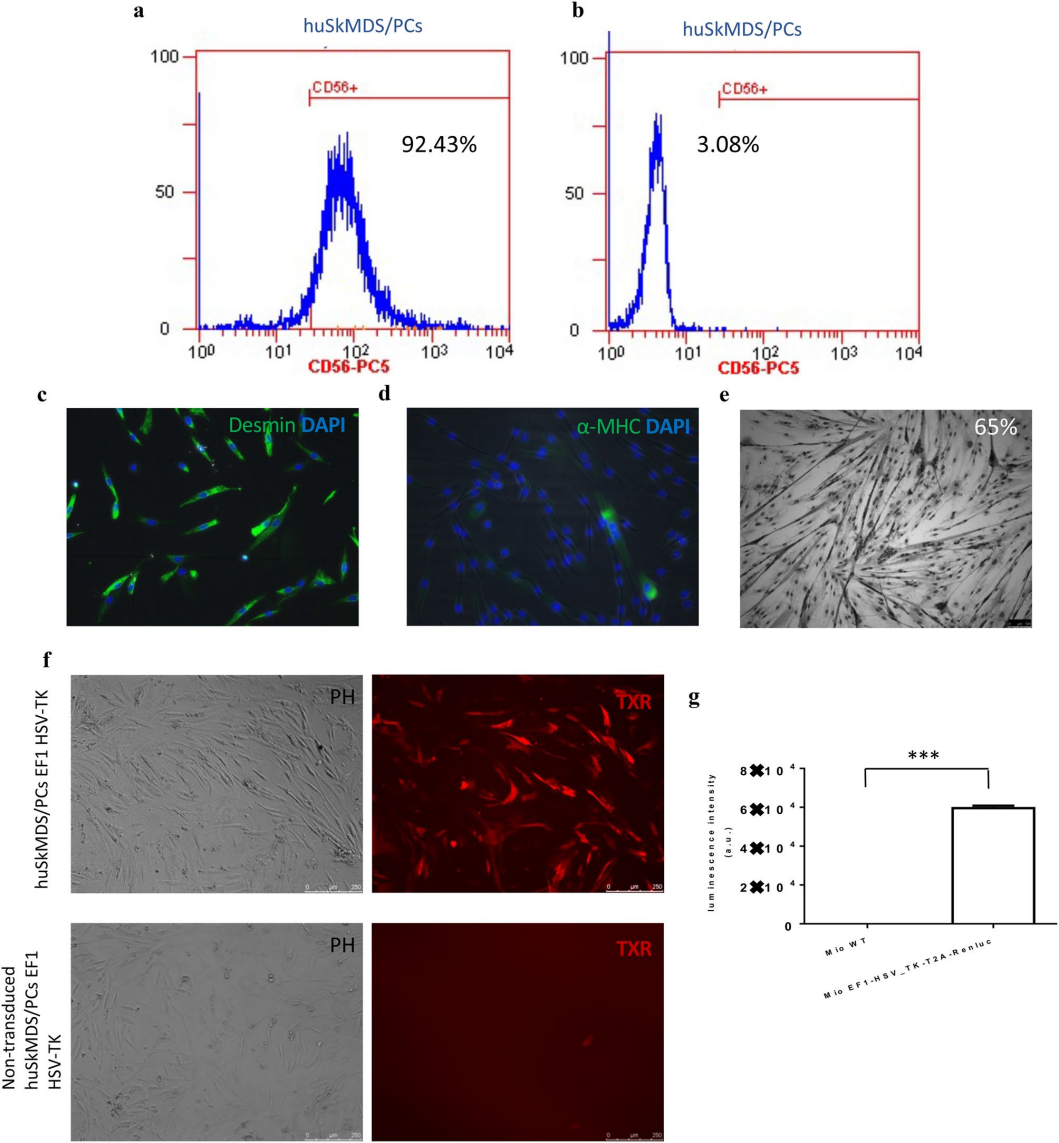
Myoblast characteristics. (**a**) Flow cytometry detected approximately 92% of CD56 + huSkMDS/PCs cells isolated from the skeletal muscle population. (**b**) Corresponding isotype control. (**c**) Immunofluorescent image of myogenic cells stained with anti-desmin antibody (green) and nuclear dye DAPI (blue). (**d**) Immunofluorescent image of myoblasts stained with anti-α-MHC (myosin heavy chain) antibody (green) with nuclear dye DAPI (blue). (**e**) Multinuclear tube formation test confirmed the ability of huSkMDS/PCs to differentiate in vitro, scale bar = 50 µm. (**f**) Positive expression of mCherry in EF1-HSV-TK transduced myogenic cells versus negative (non-transduced control), scale bar = 250 µm. (**g**) Luminescence intensity of *Renilla* measured in EF1-HSV-TK transduced huSkMDS/PCs vs. negative (non-transduced) control. Measurements were performed in triplicate for 5 × 10^4^ cells each, and p-values are given as the mean ± SD; *p < 0.05, **p < 0.01, ***p < 0.001.

Flow cytometry analysis of huSkMDS/PCs EF1-HSV-TK revealed that approximately 92% of isolated cells were CD56 + positive (Fig. 1a), with the corresponding isotype control presented in Fig. 1b. High expression of the myogenic cell marker desmin is shown in Fig. 1c, and a very low level of α-MHC (a marker of differentiated myogenic cells) is shown in Fig. 1d. The positive results of the myotube formation test showed that the cells retained their functionality in vitro (Fig. 1e). Next, huSkMDS/PCs were transduced with EF1-HSV-TK-Renluc-CMV-mCherry-T2A-PuroR lentiviral particles. To obtain a pure population of cells carrying the HSV-TK transgene, transduction was followed by selection with puromycin for 7 days. The mCherry-positive signal was observed in 95% of the cells (Fig. 1f). The statistically significant increase in *Renilla luciferase* expression in vitro in transduced cell culture vs. non-transduced cells is shown in Fig. 1g.

### Echocardiography

MI was confirmed by echocardiography by calculating the area change in the short axis (SAX AC%). There was a significant difference between MI and control mice in terms of ejection fraction volume. The MI group achieved 32.7%, and the control group reached 72.3% (Supplementary Fig. S1).

### Small-animal PET/CT imaging

#### MicroPET imaging of [^18^F]-FDG

**Figure 2:**
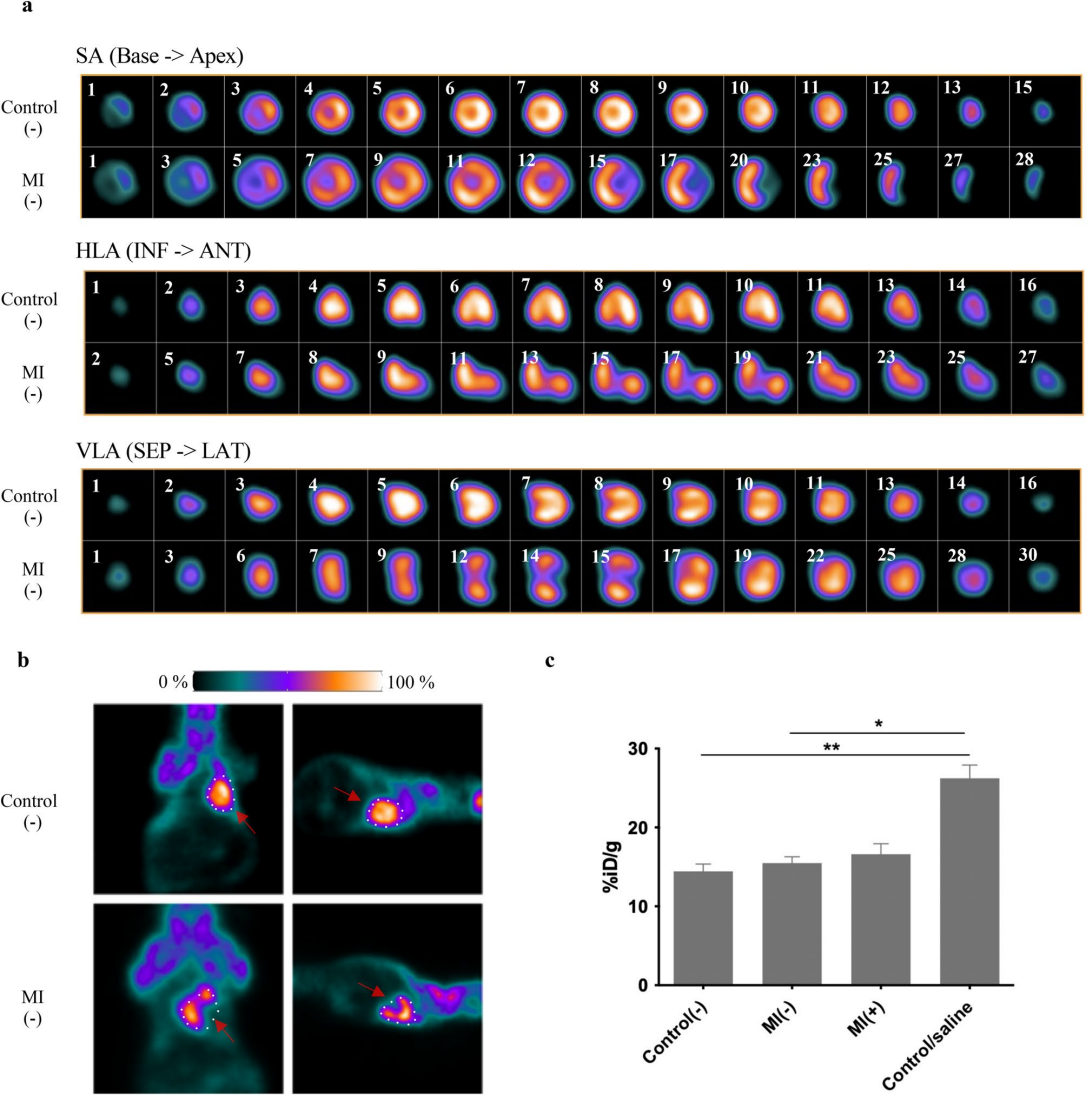
Functional and metabolic heart characteristics. (**a**) Images show a summary of cardiac viability measurements in control(−) (top row) and MI(−) (bottom row) mice administered the [^18^F]-FDG tracer. Note the presence of MI in the apex area in axial, coronal, and sagittal planes (from top to bottom) and a larger size of the post-infarction heart due to its remodelling. (**b**) Representative PET images in the dorsal and lateral positions. Red arrows show loss of uptake in apex area (**c**) Comparison of cardiac [^18^F]-FDG uptake in a group of control(−), MI(−) and MI(+) mice 7 days after cell implantation into the post-infarction heart and after saline injection. Statistical significance was evaluated with ANOVA and p-values are given as the mean ± SD; *p < 0.05, **p < 0.01.

[^18^F]-FDG metabolic marker is used to detect changes in left ventricle function after interventions in small animal models^16^. As a result of MI, metabolic and haemodynamic functions are impaired; hence, the [^18^F]-FDG indicator is potentially useful^17^. Putting together the images of control and MI hearts, we observed a difference in isotope uptake. Figure 2a presents a comparison of PET scans obtained in three planes slice by slice. The size of each heart was measured, and MI significantly influenced heart remodelling with respect to volume, revealing that post-infarction hearts were, on average, 1.8 times larger.

Using both cardiac and full scans (Fig. 2b), we noticed a significant loss of indicator in the apex region. In MI mice, both with and without cell intervention, glucose uptake was generally slightly higher than in control mice when the average value was taken into account, but for the group of mice injected with physiological saline, glucose uptake was significantly higher (Fig. 2c). SUVs for all four groups of mice are given as mean ± SD in Supplementary Fig. S2.

#### MircroPET imaging of [^18^F]-FHBG

**Figure 3:**
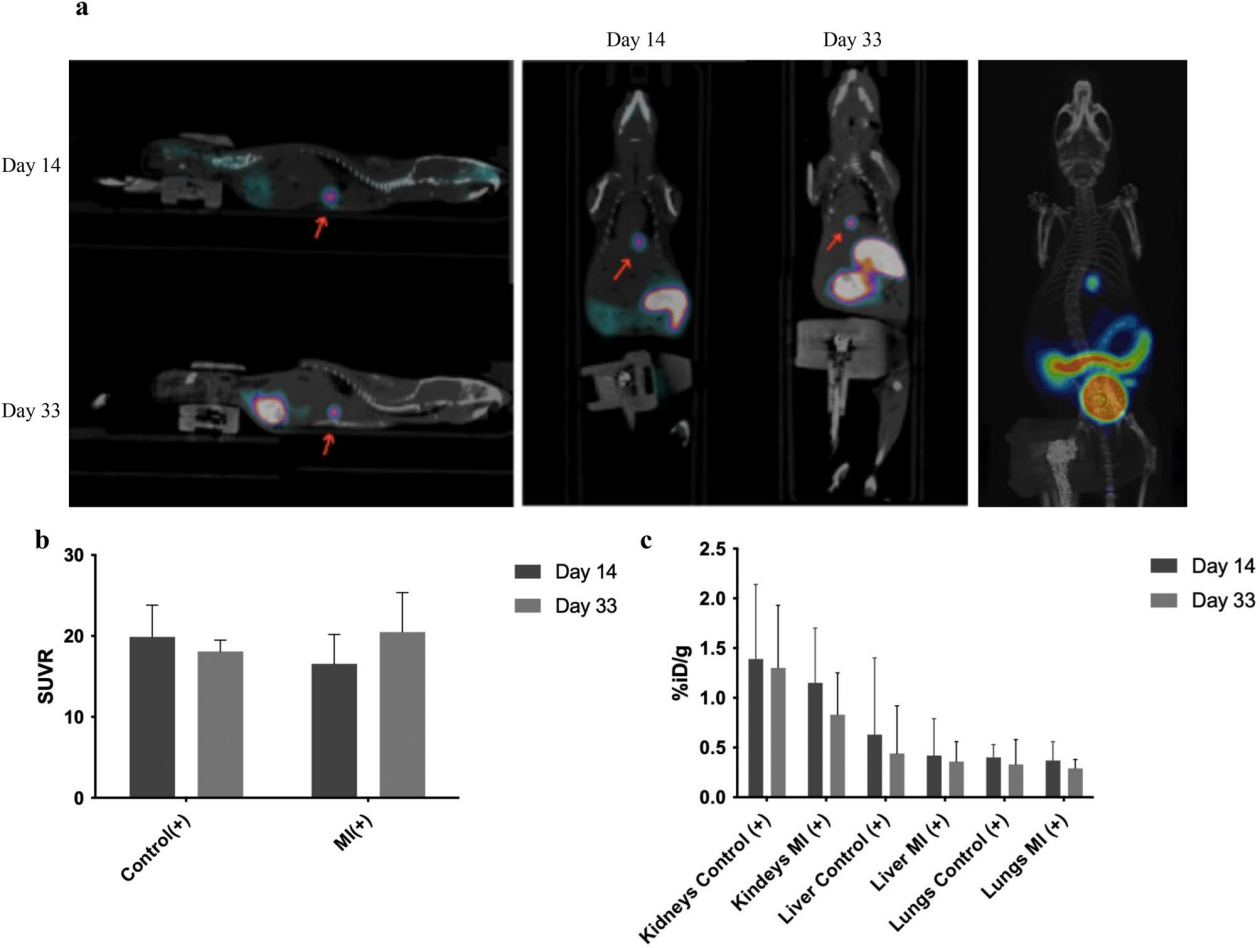
Cellular huSkMDS/PCs EF1-HSV-TK [^18^F]-FHBG PET Imaging. (**a**) The location of transplanted huSkMDs/PCs EF1-HSV-TK with [^18^F]-FHBG in the myocardium 14 and 33 days after cell transplantation (on the left) and representative 3D PET/CT image (on the right). (**b**) Comparison of cell retention in the heart over 19 days in control(+) and MI(+) mice. (**c**) Comparison of the biodistribution of the radiotracer. The bladder is detracted from the chart for better visibility.

[^18^F]-FHBG accumulates in cells when it is phosphorylated by HSV-TK, resulting trapping the compound inside the cell, where it cannot diffuse outward^18^. The fusion of PET and CT images illustrates the exact location of activity derived from [^18^F]-FHBG uptake in the apex region (Fig. 3a).

The retention of huSkMDS/PCs EF1-HSV-TK was observed in both control and MI mice. Here, we demonstrated positive imaging of huSkMDS/PCs EF1-HSV-TK in the mouse myocardium within a month of cell implantation. The cell retention in control and MI hearts varied; however, the radiotracer uptake did not fall below 50% (Fig. 3b).

VOIs were set around the liver, kidney, lung, and bladder in the scans to determine accumulation of the compound in the solid organs (Fig. 3c). The compound primarily accumulated in the bladder and intestines (Fig. 3a). Increased kidney accumulation was observed due to the vicinity to the gut, while increased hepatic values may be due to compound accumulation in huSkMDS/PCs EF1-HSV-TK around the apex area of the heart. Note the chemical purity of the compound and the lack of free fluorides in systemic fluids compared to those tested so far^9^.

SUVs were calculated based on manually drawn regions as previously described^19^. SUVs are shown as the mean ± SD for heart, kidney, lung, liver, and bladder in Supplementary Fig. S3.

#### Myocardial viability with [^18^F]-FDG

**Figure 4:**
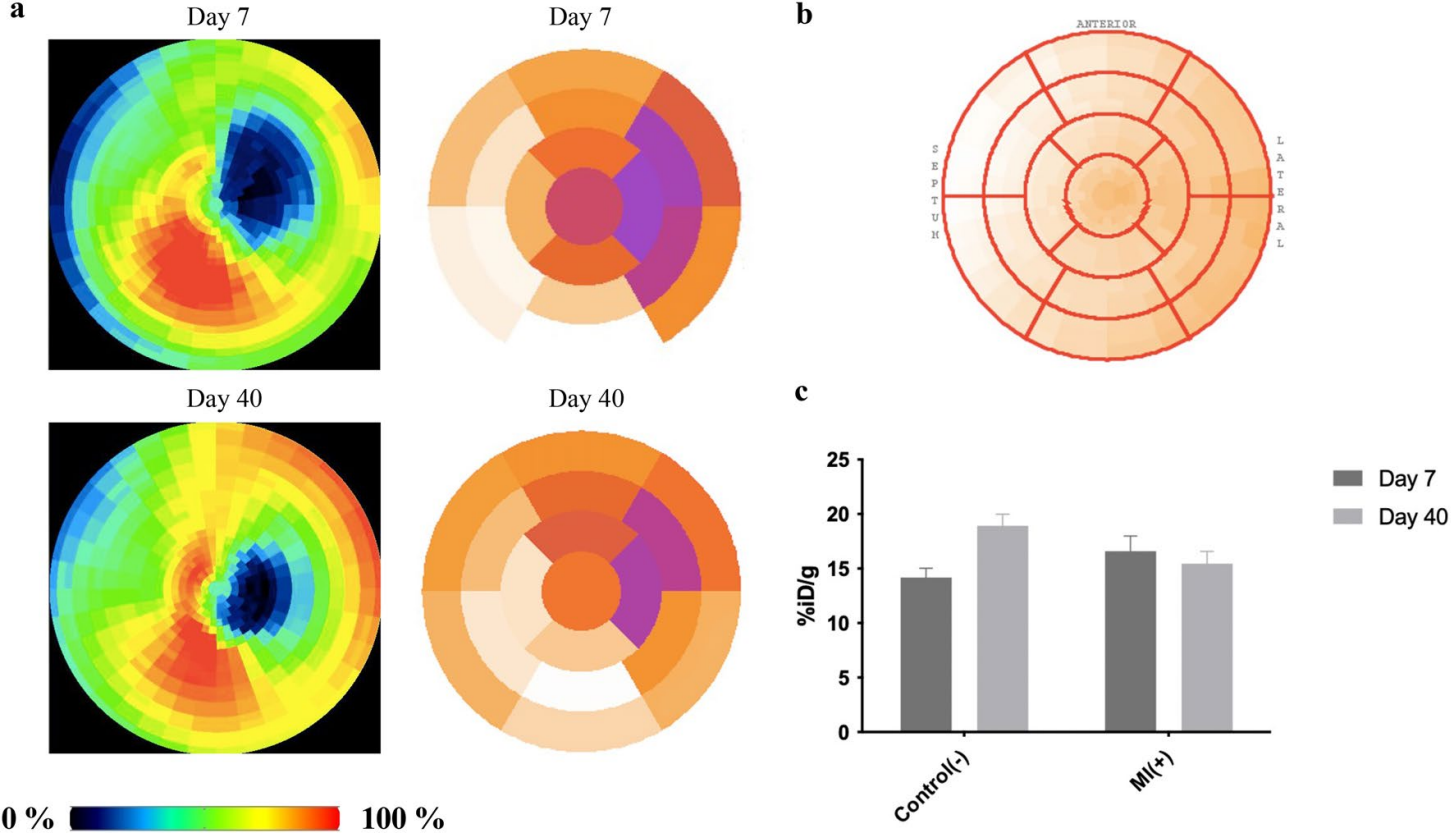
Metabolic heart activity (viability) measured through glucose uptake. (**a**) Conversion of PET images to maps of radiotracer activity in the heart (left) and their counterparts presented as polar maps for MI( +) mice (right). A reduction in the metabolically inactive zone can be seen in the walls and apex segments. (**b**) Scheme of the polar map by 17 AHA segmentation with indication of septum, anterior and lateral directions. (**c**) Graph for dual cardiac viability PET imaging in control(−) and MI( +).

Cardiac PET provides high sensitivity for the examination of regional changes in myocardial metabolism^20^. We performed double imaging using [^18^F]-FDG. By obtaining polar maps, it was possible to show not only the location but also the size and severity of metabolic dysfunction of individual segments^21^ according to 17 American Heart Association (AHA)^22^. Figure 4a shows marker accumulation in the MI group compared to the control heart (Fig. 4b). The areas inactive in the predicted heart segments can thus be identified. In working segments, accumulation was higher than in control mice. PET images were assembled as polar maps as mentioned previously^23^. MI mice exhibited 3 ± 1 damaged segments after surgery, corresponding to 18% of the myocardium. Control mice showed no loss of isotope accumulation (Fig. 4b).

Dual imaging visualized the reduction of metabolically inactive zone following cell therapy; however, tracer uptake remained constant and did not differ significantly from uptake in control hearts (Fig. 4c). SUVs are given as the mean ± SD for dual cardiac viability PET imaging in Supplementary Fig. S4.

### Improvement of cardiac viability determined by MRI imaging

**Figure 5:**
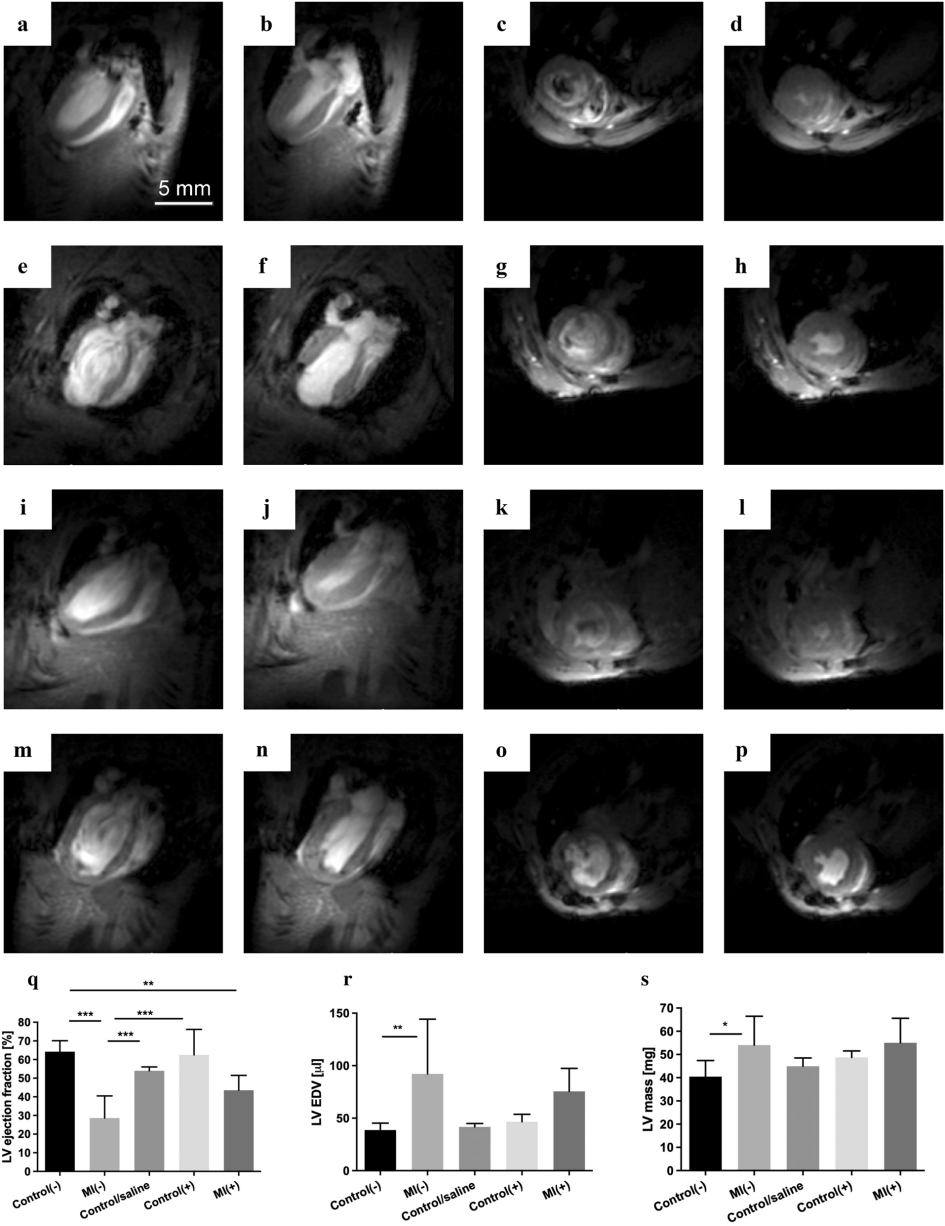
Representative images of end-diastolic and end-systolic volumes from the short and long axes. (**a**–**d**) control(−), (**e**–**h**) MI(−), (**i**–**l**) control(+), and (**m**–**p**) MI(+) hearts. (**q**) Comparison of LV ejection fraction, (**r**) Comparison of LV EDV volumes, (**s**) Comparison of LV mass. Statistical significance was evaluated with ANOVA and p-values are given as the mean ± SD; *p < 0.05, **p < 0.01, ***p < 0.001.

MRI showed improvement in LV function following cell intervention. End-diastolic and end-systolic volumes from two short- and long-axis images were obtained from 4 groups of mice: control(−), MI(−), control(+), and MI(+) (Fig. 5a–p), as well as from mice with saline injected intracardially (Supplementary Fig. S5). We present the obtained parameters in Fig. 5r–t. The LV ejection fraction significantly decreased in MI(−) mice, while the LV EDV parameter and LV mass increased compared to the other groups. In MI(+) mice treated with applied cell therapy, functional haemodynamic improvement was observed compared to MI(−) alone. The administration effect itself of physiological saline in control/saline group seemed to influence the haemodynamic parameters of the heart, justifying the certain delay in molecular testing after cell intervention (Supplementary Fig. S6).

## Discussion

MI is one of the greatest threats to human life and can result from many comorbidities or risk factors. MI rapidly causes cardiac cell death and reduces organ functionality^24^. A non-contractile scar impairs normal blood flow^25^ and glucose oxidative metabolism^26^, inducing heart remodelling. The function of individual segments of the heart is impaired with time, which may cause a cascade of further pathogenic events. Segment inactivity, following MI, influences heart wall structure and increases the risk of heart failure^27^. Leaving damaged cardiac tissue without intervention often leads to repeated infarctions and sudden deaths, which are at high levels during the first months after MI, among patients with low LV ejection fraction^28^. The revascularization procedure applied after MI does not necessarily restore the original structure and capacity of the myocardium without additional procedures^29^. The challenge is to restore the original heart and normal cardiomyocyte functions.

There is a link between the presence of transplanted stem cells and a role in natural organ repair. While stem cell grafts have been shown to exert a positive effect on the restoration of functional myocardial tissue, little is known about stimulating the heart to regenerate itself after MI. Heart cellular therapies replenish the post-infarction scar with the intention of restoring original contractility and viability^30,31^. Myogenic cells are usually isolated from skeletal muscle biopsies and then expanded in vitro for subsequent grafting^32^. Moreover, the precise determination of therapeutic effects, the assessment of colonization, and cell survival remain elusive. Therefore, it is necessary to develop alternative monitoring methods that allow a non-invasive assessment of the regenerative effect of cells on the post-infarction myocardium in preclinical models^33^. To ensure effective regeneration, one should focus on two stages: selecting the optimal source of candidate cells that best reflects the work of cardiomyocytes and monitoring their activities in situ (retention, biodistribution, functional effect).

Optical techniques are gaining popularity in non-invasive imaging, such as BLI (e.g., IVIS Lumina). However, this type of imaging is limited to small laboratory animals^34^. To perform long-term imaging, in future clinical trials, we need more advanced procedures suitable not only for rodents but also for larger animal imaging.

The purpose of this study was to achieve molecular imaging of implanted genetically modified stem/progenitor cells with a gene reporter system that is visible in the post-infarction animal myocardium. We described the use of [^18^F]-FHBG as a molecular reporter probe for huSkMDS/PCs EF1-HSV-TK in vivo tracking in a living post-infarction mouse model. MicroPET imaging of animals carrying huSkMDS/PCs EF1-HSV-TK revealed visible, highly specific radiotracer retention in the periapical region (Fig. 3a). Total cell radioactivity only includes phosphorylated labels, which can be associated with the expression of the HSV1-TK reporter gene. Radionuclide [^18^F]-FDG was used to control cardiac viability mechanisms that rely on glucose uptake and showed a metabolic defect (Fig. 4a). This method illustrates the use of glucose metabolism to ascertain the localization and size of the infarction zone. In addition, it can be concluded that [^18^F]-FDG enables the monitoring of therapeutic outcomes, and the marker [^18^F]-FHBG reporter provides quantitative and qualitative information during cellular therapy^35^. We also used an intracardiac administration mimic by intracardial delivery of saline alone. This procedure provided information that allowed us to postpone [^18^F]-FDG imaging of the mice immediately after the transplant, which made it possible to remove false radiotracer uptake caused by inflammation in the heart directly after injection.

Herein, we present proof of a functional improvement in the peri-infarction zone by reducing the infarct size (Fig. 4a). Moreover, the presence of huSkMDS/PC EF1-HSV-TK transplants showed a potential haemodynamic effect at improving the myocardial function of the left ventricle (Fig. 5r–t). Cell retention in the heart may be a key point in regenerating the MI myocardium and restoring patient quality of life. MRI and PET measurements confirmed the improvement of left ventricular viability after cell intervention and demonstrated improvement of haemodynamic parameters. Additional studies are currently underway to determine the retention efficiency of FHBG in vitro over time. In the future, it is worth considering additional IHC analysis of mouse hearts to reinforce research to confirm the exact location of the cells. Additionally, mCherry flow cytometry analysis can be performed to indicate cell frequency, but our instruments did not have this function. Taken together, we demonstrated the possibility of non-invasive molecular imaging protocols examining the regenerative process in the myocardium in response to cellular therapy.

The purpose of this study was to enable long-term imaging of transplanted genetically modified stem/progenitor cells in the post-infarction small animal myocardium. We described the use of [^18^F]-FHBG as a reporter probe for HSV1-TK in vivo in living healthy and MI mice to monitor implanted cells. MicroPET imaging of mice carrying huSkMDS/PC EF1-HSV-TK cells revealed visible, highly specific radiotracer retention, while [^18^F]-FDG PET/CT and MRI imaging confirmed improvement of cardiac function as a result of cellular therapy.

## Materials and methods

### Animal model

Experiments were performed in 3-month-old male and female non-obese diabetic/severe combined immunodeficiency mice (NOD, CB17-*Prkdc*^*scid*^/NCrCrl, Charles River). NOD-SCID mice tolerate experimental conditions, xenogeneic cells, and tissues, making them an excellent experimental model for the study of myocardial physiology and molecular imaging of human cells. PET/CT imaging was performed on five groups of mice: healthy mice (control(−), n = 8), healthy + huSkMDS/PCs EF1-HSV-TK mice (control(+), n = 4), MI mice (MI(−),n = 6), MI + huSkMDS/PCs EF1-HSV-TK mice (MI(+)n = 5), and healthy mice intracardially administered physiological saline (control/saline, n = 7).

### Experimental design

Control echocardiography was performed, and then MI was induced by left coronary artery ligation on the day set as “− 22”. To confirm MI, a second echocardiography was performed (day -6). On the day assigned as “0” huSkMDS/PC EF1-HSV-TK cells were implanted intramyocardially into two groups of mice, control(+) and MI(+). Next, we monitored the metabolic function of the saline-injected mice (control/saline) to check the impact of the cardiac injection, then we did the research on control(−) and MI(+) groups using [18F]-FDG PET/CT measurements (days 7 and 40). In MI mice, first [18F]-FDG measurement was performed 30 days following MI. Between [^18^F]-FDG measurements, long-term cell imaging with [^18^F]-FHBG was performed at 14 and 33 day following cell transplantation. MRI preceded animal euthanasia accordingly. The experimental design is presented in Supplementary Fig. S7.

### huSkMDS/PCs in vitro cell culture and characteristics

huSkMDS/PCs were isolated from remaining tissue fragments after the anterior cruciate ligament (ACL) surgical procedure and were cultured in vitro as previously described^36^. The volunteer was a 33-year-old male. Written informed consent was obtained from the study participant for the tissue donation and all the procedures, including protocols based on recommendations for human tissue collection from the Local Ethical Committee, University of Medical Sciences, Poznan. We confirm that all methods used were performed in accordance with the relevant guidelines and regulations. At the same time, we should like to assure that all the experimental protocols used in this study were approved by the Local Bioethical Committee, Poznan, Poland. The tissue fragment was mechanically dissected using scalpel blade and subjected to digestion (0.02% collagenase solution). The obtained cell suspension was filtered (trough 80-µm mesh) and centrifuged. Next, cell suspension was plated on 0.1% gelatine-covered culture dish. Cells were cultured in standard Dulbecco’s modified Eagle medium with 4.5 g/l glucose (Lonza, Basel, Switzerland) supplemented with 20% foetal bovine serum (Lonza, Basel, Switzerland), 1% penicillin/streptomycin (Lonza, Basel, Switzerland), 1% ultraglutamine (Lonza, Basel, Switzerland), 1% chicken embryo extract (Sera Laboratories International, West Sussex, UK) and bFGF (Sigma-Aldrich, St. Louis, MO, USA) under standard culture conditions (95% humidity, 5% CO_2_, at 37 °C). The medium was changed every other day. To avoid spontaneous myotube formation, the cells were passaged every 4–5 days (at 70% confluency) using 0.25% trypsin and EDTA solution (Lonza, Basel, Switzerland). Respective marker cell immunostaining, flow cytometry, and multinuclear tube formation, according to the protocols described by Fiedorowicz^37^, confirmed the myogenic characteristics of the cells.

### Immunofluorescence staining of in vitro cultured huSkMDS/PCs

Myogenic cell markers were confirmed by immunofluorescence using the anti-desmin and anti-MHC antibodies listed in Supplementary Table S1.

Prior to immunofluorescence, paraformaldehyde cell fixation (4% paraformaldehyde solution in PBS) was performed. Fixation was followed by three washes in PBS. For permeabilization, cells were incubated in 0.1% Triton-X-100 (Sigma-Aldrich, St. Louis, MO, USA) for 15 min. Next, cells were preincubated with 10% goat serum diluted in PBS with 0.1% Triton X-100/PBS solution to block nonspecific epitopes for an additional 60 min at room temperature. After removal of the blocking serum, cells were incubated overnight at 4 °C with primary antibody diluted in PBS and 0.1% Triton X-100. The secondary antibody conjugated with a fluorochrome was added for 60 min. After three washes in PBS, DAPI (Sigma-Aldrich, St. Louis, MO, USA) was added to visualize the cell nuclei. Stained preparations were observed under Leica DMi8 and an Olympus BX40 fluorescence microscope.

### Flow cytometry analysis

The purity of the cell population was evaluated using an anti-CD56-antibody-PC5 conjugate (Beckman Coulter, Inc. Brea, CA, USA) by flow cytometry (Beckman Coulter, Inc. Brea, CA, USA). Briefly, 0.25 × 10^6^ cell aliquots were harvested, centrifuged (1200 rpm, 10 min), resuspended in 100 µl of phosphate-buffered saline (PBS) with 2% FBS and incubated with 10 µl of an anti-CD56 antibody or the respective isotype control at a 1:200 dilution.

### Multinuclear tube formation

To perform the multinuclear tube formation functional test, cells were differentiated in medium consisting of Dulbecco’s modified Eagle’s medium containing 4.5 g/l glucose (Lonza, Basel, Switzerland) supplemented with 2% horse serum (Lonza, Basel, Switzerland), 1% penicillin/streptomycin (Lonza, Basel, Switzerland), and 1% ultraglutamine (Lonza, Basel, Switzerland) under standard culture conditions (95% humidity, 5% CO_2_, and 37 °C) for at least 7 days. The percentage of cells with 2 or more nuclei was assessed.

### Generation of EF1-HSV-TK-T2A-Renluc-CMV-mCherry-T2A-puroR huSkMDS/PC cell suspension

The EF1-HSV-TK-Renluc-CMV-mCherry-PuroR vector was prepared by Vector Builder (VectorBuilder Inc., TX, USA). The EF1 promoter drove overexpression of HSV-TK and *Renilla luciferase* (Supplementary Fig. S8).

In turn, the CMV constitutive promoter controlled mCherry overexpression and puromycin resistance genes. Lentiviral particles were produced using a second generation packaging system. An hour before transduction, huSkMDS/PCs were treated with polybrene (Millipor Polybrene Infection/Transfection Reagent, 5 µg/ml). The huSkMDS/PCs with medium containing viral particles carrying the EF1-HSV-Renluc-CMV-mCherry-PuroR transgene supplemented with polybrene (5 µg/ml) were incubated for 24 h. Afterward, the procedure with medium containing viral particles was repeated. We selected a population of cells carrying the reporter gene using puromycin (0.3 µg/ml for 7 days) and observed expression of mCherry in selected cells under a fluorescence microscope (Leica DMi8). The luminescence was measured using the Pierce *Renilla Luciferase* Flash Assay Kit (Thermo Scientific, Waltham, MA, USA). Measurements were performed in triplicate (5 × 10^4^ cells) for non-transduced and EF1-HSV-TK-transduced cells using a GloMax luminometer (Promega, Madison, WI, USA).

### Left ventricular functional analysis and echocardiographic evaluation of MI

To assess cardiac parameters, echocardiography was performed and evaluated as described by Wiernicki et al.^38^, with the exception that for animal anaesthesia, 2% isoflurane/oxygen was used.

### MI and stem/progenitor cell delivery

On the day assigned as “− 22”, mice were initially anaesthetized with isoflurane (4%), intubated, and then kept under isoflurane anaesthesia (2%) and ventilation with a mix of oxygen. Surgery was performed by ligating the left anterior descending artery (LAD) with a suture.

Intramyocardial injection of 1.5 × 10^6^ huSkMDS/PCs EF1-HSV-TK cells in 30 µl volume in each animal into the peri-infarction zone was performed on day “0” following MI induction under the same anaesthesia conditions. We performed parallel cellular intervention in a control group.

### Small-animal PET/CT imaging

#### Radiolabelled compounds

Fluorine-18 was obtained from VOXEL S.A. (Cracow, Poland) with radiochemical purity > 99.9%. The input activity per synthesizer was 10 GBq. Product quality control comprised testing radiochemical and chemical purity. High-performance liquid chromatography (HPLC) (Shimadzu AD 20 HPLC with UV–Vis detector) with a radiometric detector (GabiStar, Raytest, Germany) was used to determine these parameters. During HPLC analysis, a C18-RP (Phenomenex Gemini C18 150 mm × 4.6 mm × 5 µm) column was used. The Atomlab 500 (Biodex Medical Systems, Shirley, NY, USA) dose calibrator was used for all activity measurements. 9-(4-18F-fluoro-3-[hydroxymethyl]butyl)guanine was synthesized at the University of Warsaw, Biological and Chemical Research Centre. We obtained the product with a radiochemical purity > 98.5% (Supplementary Fig. S9).

#### System

In the following work, we used a tri-modal small-animal scanner Albira Si PET/SPECT/CT Preclinical Imaging System (Bruker, Billerica, MA, USA). The Albira Si PET/SPECT/CT system provides high-resolution PET imaging with automated CT image fusion for anatomical reference^39^.

#### Acquisition protocol [^18^F]-FDG and [^18^F]- FHBG

On the 7th day, four groups were injected with [^18^F]-FDG (11.75 ± 1.67 MBq) via the tail vein in a total volume of 150 µl in each animal. On days 14 and 33 after cell transplantation, two groups with cell intervention were administered [^18^F]-FHBG (5.15 ± 1.37 The PET/CT scan started 60 min after isotope administration. Mice were placed in an induction chamber for initial anaesthesia (isoflurane 3.5–4%). During the imaging procedure, we kept the animals under general anaesthesia (isoflurane 1.5–2%). In the case of cardiac viability in the control(−) and MI(+) groups, the measurements were repeated at the appropriate time.

#### PET/CT image fusion and data analysis

PET imaging data were reconstructed using the built-in program Albira reconstruction software and analysed in PMOD v4.02. To define the site of the reporter probe [^18^F]-FHBG and metabolic marker (FDG accumulation in anatomical visualization), colour-coded PET images were superimposed on inverted greyscale CT images. The reorientation parameters were set by the software, so any mismatch of image fusion must be corrected manually during image analysis. Three-dimensional regions of interest (ROIs) were drawn manually over the isotope uptake areas in the heart. Additionally, to calculate the volume of each heart, ROIs were drawn over healthy and MI hearts. Mean SUV and SUVR calculations were based on set ROIs in the heart and as a reference region for SUVR we have used pulmonary activity uptake.

### MRI acquisition

Magnetic resonance imaging (MRI) was performed to assess cardiac functional parameters: ejection fraction and end-diastolic volume of both ventricles. All data were collected using a 7 T Bruker Biospec scanner (70/30 USR, Bruker Biospin, Ettlingen, Germany). A receiver-only surface coil (10 mm inner diameter) and transmit cylindrical radiofrequency volume coil (8.6 cm inner diameter) were used in the experiment. Mice were anaesthetized and placed in the MR-compatible animal bed. Based on pilot scans, a 4-chamber view on a long heart axis was acquired. It has been used for setting geometry for a package of short-axis scans that covered all ventricle volumes. These images were acquired with the IntraGateFLASH protocol using the following parameters: echo time TE = 3 ms, repetition time TR = 10 ms, number of repetitions NR = 120, field of view FOV = 25 mm × 25 mm, slice thickness = 0.9 mm, and spatial resolution = 0.13 mm × 0.13 mm for pixels. Fifteen images per heartbeat cycle were acquired, and a gating system with manually set heart and respiration rates was used.

### MRI data analysis

Acquired data were reconstructed in DICOM format using the Paravision system provided by Bruker. For each short-axis scan, there was a set of images representing a heartbeat cycle. End-diastolic and end-systolic images have been established. For these images, the inner edges of both ventricles were manually outlined. Repeating the process on each slice allowed calculation of cardiac haemodynamic parameters: ejection fraction and end-diastolic volume of both ventricles.

### Statistics

All data are presented as the mean values ± standard deviation (SD). Statistical significance was evaluated with Mann–Whitney, ANOVA and Student’s *t*-test. Values of *p* < 0.05 were considered statistically significant. Statistical analysis was performed using GraphPad v5.01 (GraphPad Inc., LA Jolla, CA, USA).

### Ethical approval

All applicable international, national, and/or institutional guidelines for the care and use of animals were followed. Study protocols were approved by the Local Ethical Committee of the Poznan University of Life Sciences. The experiments complied with the ARRIVE guidelines^40^.

## Supplementary Information

Supplementary Information.

## Data Availability

The datasets generated during and/or analysed during the current study are available from the corresponding author on reasonable request.

## Abbreviations

[^18^F]-FDG: 2-Deoxy-18F-fluorodeoxyglucose
[^18^F]-FHBG: 9-(4-18F-Fluoro-3-[hydroxymethyl]butyl)guanine
CT: Computed tomography
HSV-TK: Herpes simplex virus thymidine kinase
huSkMDS/PCs: Human skeletal muscle derived stem/progenitor cells
LAD: Left anterior descending artery
LV: Left ventricle
MI: Myocardial infarction
MRI: Magnetic resonance imaging
PET: Positron emission tomography
SUV: Standardized uptake value
SUVR: Standardized uptake value with reference
VOI: Volume of interest
