# Supplementary material for: Molecular imaging of myogenic stem/progenitor cells with [18F]-FHBG PET/CT system in SCID mice model of post-infarction heart
Source: Sci Rep. 2021 Oct 6;11:19825. doi: 10.1038/s41598-021-98861-5 (PMC8494811; doi:10.1038/s41598-021-98861-5)

Molecular imaging of myogenic stem/progenitor cells with [^{18}F]-FHBG PET/CT system in SCID mice model of post-infarction heart.

European Journal of Nuclear Medicine and Molecular Imaging

Weronika Wargocka-Matuszewska [1], Katarzyna Fiedorowicz [2], Anna Rugowska [3], Karolina Bednarowicz [2], Agnieszka Zimna [2], Łukasz Cheda [1], Paulina Hamankiewicz [1], Krzysztof Kilian [4], Michał Fiedorowicz [5], Monika Drabik [5], Natalia Rozwadowska [2], Zbigniew Rogulski [1*], Maciej Kurpisz [2*]

[1] Faculty of Chemistry, Biological and Chemical Research Centre, University of Warsaw, Żwirki i Wigury 101, 02-089 Warsaw, Poland

[2] Institute of Human Genetics Polish Academy of Science, Strzeszyńska 32, 60-479 Poznań, Poland

[3] Institute of Human Biology and Evolution, Faculty of Biology Adam Mickiewicz University, Uniwersytetu Poznańskiego 6, 61-614 Poznań, Poland

[4] Heavy Ion Laboratory University of Warsaw, Pasteura 5A, 02-093 Warsaw, Poland

[5] Mossakowski Medical Research Centre Polish Academy of Science, Pawińskiego 5, 02-106 Warsaw, Poland

* Corresponding authors:
Zbigniew Rogulski
Faculty of Chemistry, Biological and Chemical Research Centre, University of Warsaw, Żwirki i Wigury 101, 02-089 Warsaw, Poland
ORCID ID 0000-0002-2372-850X
rogul@chem.uw.edu.pl

Maciej Kurpisz
Institute of Human Genetics Polish Academy of Science, Strzeszyńska 32, 60-479 Poznań, Poland
ORCID ID 0000-0003-3275-3245
maciej.kurpisz@igcz.poznan.pl

Supplementary Fig. S1 Comparison of SAX/END in control and post-infarction mice

Measurements were taken before induction of myocardial infarction and 17 days after the formation of the post-infarction scar, and p-values are shown as the mean ± SD; *p < 0.05, **p < 0.01, ***p < 0.001.

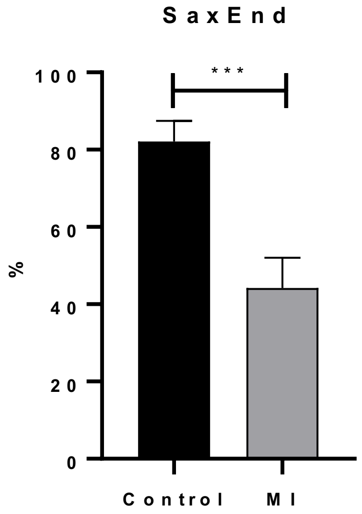

Supplementary Fig. S2 Functional and metabolic heart characteristics

Standard uptake values of all four groups of mice. SUVs are given as mean +/- SD.

	%iD/g	
Mice	SUV	SD
Control(-)	14,45	0,92
MI(-)	15,48	0,8
MI(+)	16,60	1,33
Control/saline	26,24	1,67

Supplementary Fig. S3 Cellular huSkMDS/PCs EF1-HSV-TK [^{18}F]-FHBG PET Imaging

Standard uptake values of two groups of mice. SUVs are shown as the mean +/- SD for heart, kidney, lung, liver, and bladder.

		Heart		Kidneys		Liver		Lungs		Bladder	
Mice	Day	SUV	SD	SUV	SD	SUV	SD	SUV	SD	SUV	SD
Control(+)	Day 14	7,95	0,51	1,39	0,75	0,63	0,77	0,40	0,13	438,43	36,64
	Day 33	5,97	0,35	1,01	0,65	0,44	0,48	0,33	0,25	270,65	25,72
MI(+)	Day 14	6,13	0,69	1,15	0,55	0,42	0,37	0,37	0,19	287,73	33,24
	Day 33	5,94	0,44	0,83	0,42	0,36	0,20	0,29	0,09	119,77	10,04

Supplementary Fig. S4 Metabolic heart activity (viability) measured through glucose uptake

Standard uptake values for dual cardiac viability PET imaging in of two groups of mice. SUVs are shown as the mean +/- SD.

		%iD/g	
Mice	Day	SUV	SD
Control(-)	Day 7	14,17	0,87
	Day 40	18,92	1,06
MI(+)	Day 7	16,60	1,38
	Day 40	15,44	1,14

Supplementary Fig. S5 Representative images of end-diastolic and end-systolic volumes from the short and long axes of mice intracardially injected with saline.

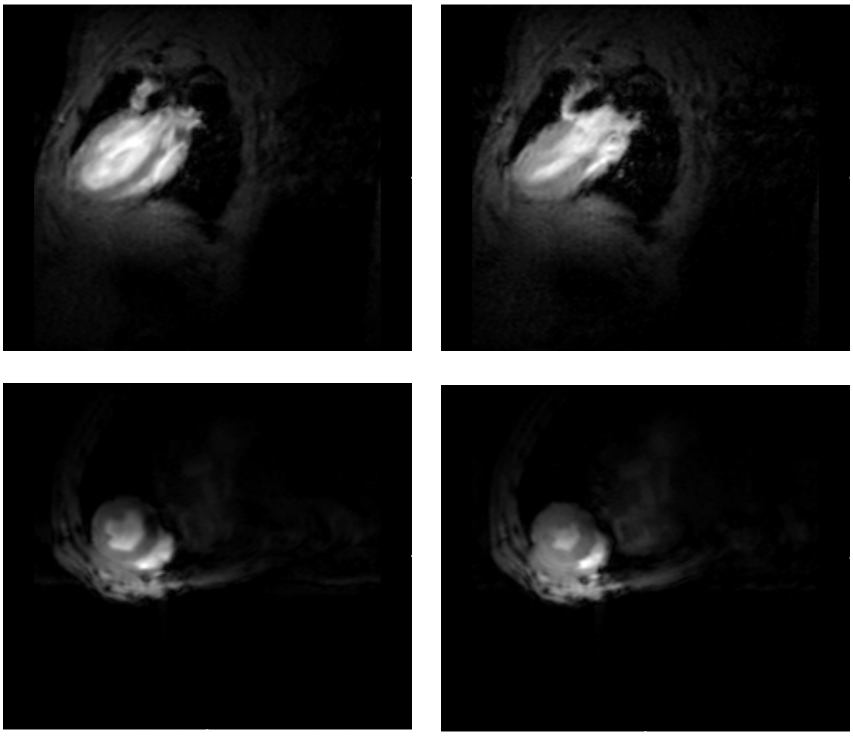

Supplementary Fig. S6 Haemodynamic analysis of intact control hearts and after saline administration. The results are shown as the mean +/- SD; * p <0.05; ** p <0.01 Mann-Whitney U test.

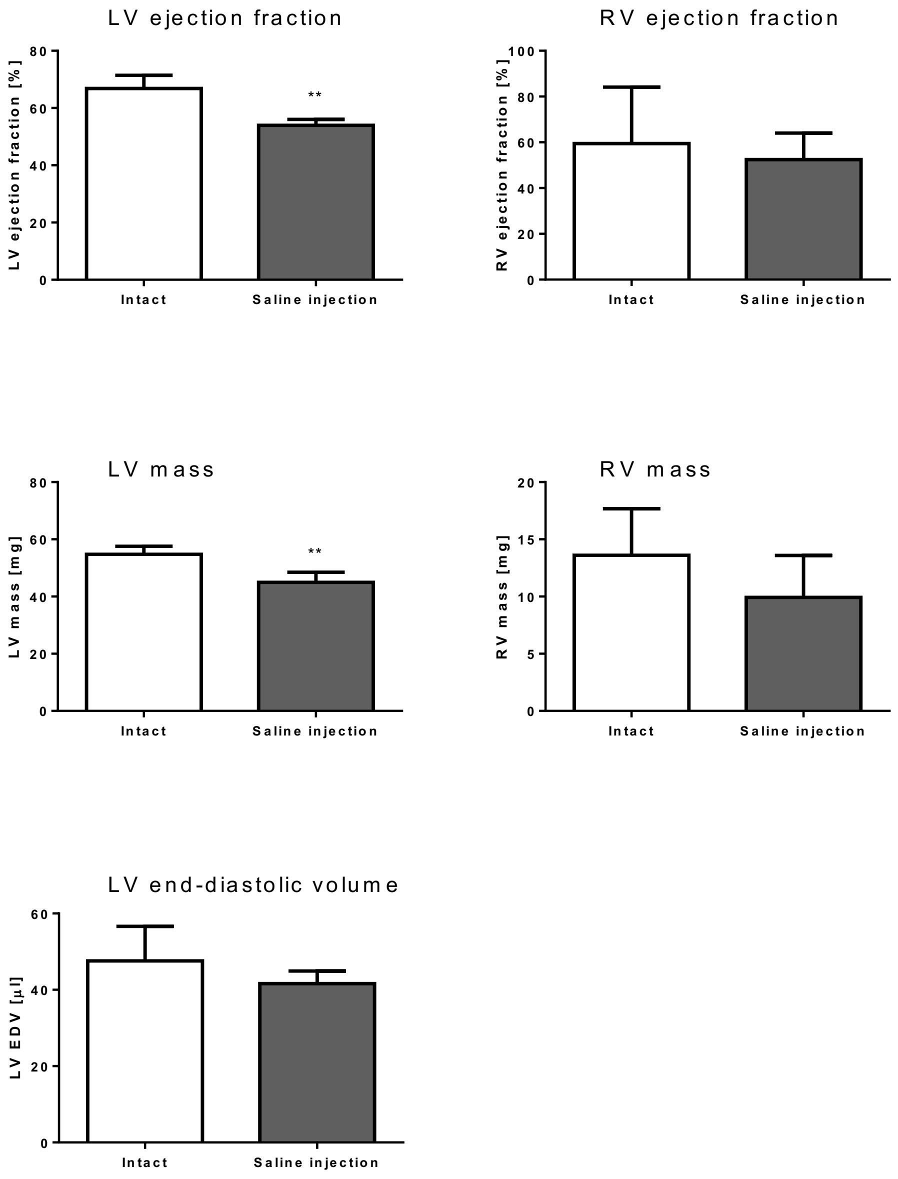

Supplementary Fig. S7 Experimental design

The graph shows all stages of the experimental procedures included in the study of the post-infarction mouse model with huSkMDS/PC EF1-HSV-TK interventions.

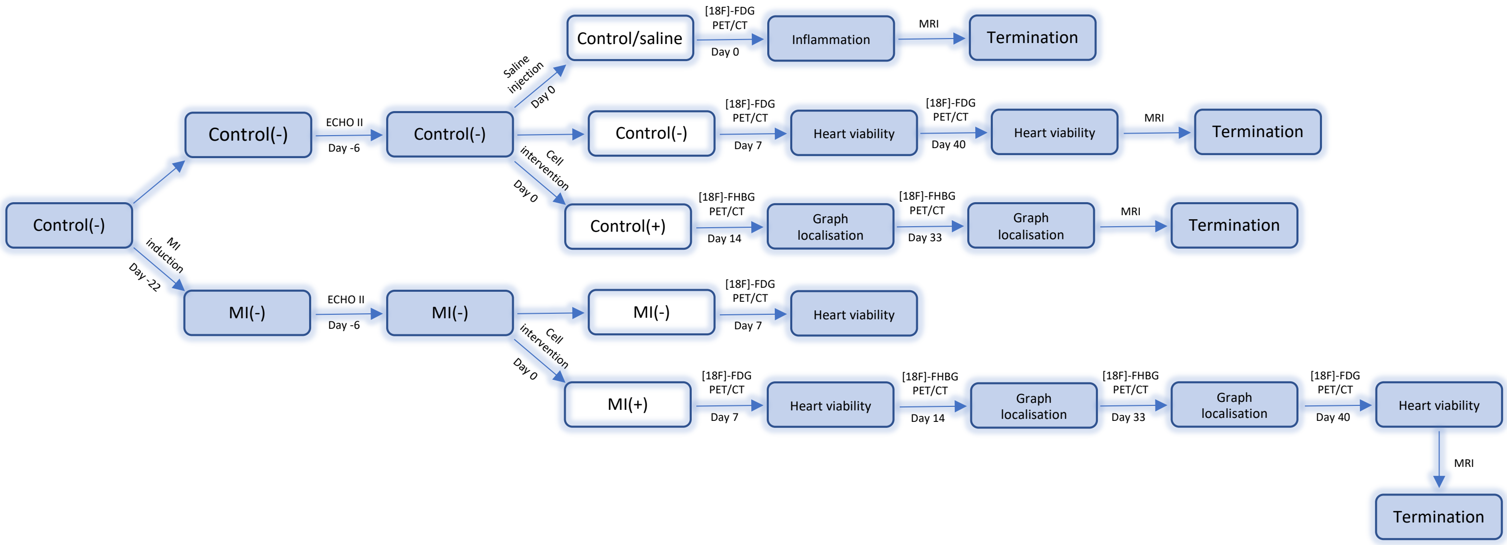

Supplementary Table S1 Primary and secondary antibodies used for immunofluorescence

Antibody	Manufacturer	Characteristics	Dilution
mouse anti-desmin	Abcam Cambridge (UK)	Myogenic markers	1:200
mouse anti-heavy chain myosin			1:400
anti-mouse Alexa Fluor 488	Abcam Cambridge (UK)	Flurochrome conjugated secondary antibody	1:500

Supplementary Fig. S8 Construction of lentiviral vector

EF1-HSV-TK-*renluc-CMV-mCherry-PuroR: Renilla luciferase* (Renluc) and thymidine kinase (HSV-TK) expression is controlled by constitutively expressed promoter, elongation factor 1 (EF1), mCMV constitutive promoter controls expression of mCherry and puromycin resistance (PuroR).

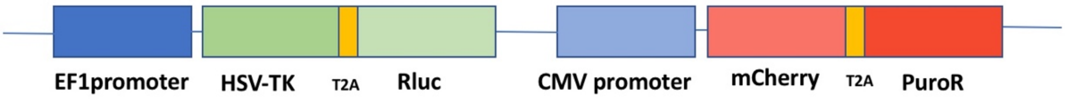

Supplementary Fig. S9 Quality control of [^{18}F]-FHBG (RadioHPLC). Chromatogram: 1 - fluorine-18, 2 - [^{18}F]-FHBG.

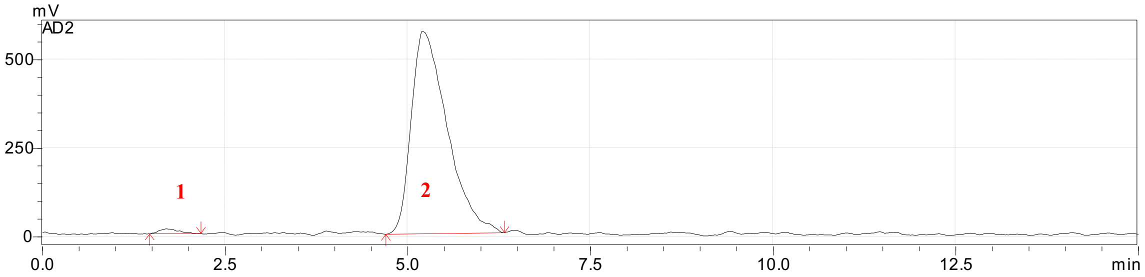

Supplement: Supplementary file 1 — Supplementary Information. [file 41598_2021_98861_MOESM1_ESM.pdf]
